# A case of pyothorax after treatment of burn inhalation injury. A case report

**DOI:** 10.1016/j.ijscr.2023.109048

**Published:** 2023-11-20

**Authors:** Asuka Uebayashi, Toshinari Ema, Hiroaki Oiwa, Syo Morita, Hiroaki Sakai, Kazuhito Funai

**Affiliations:** a Department of General Thoracic Surgery, Fujieda Municipal General Hospital, 4-1-11, Surugadai, Fujieda-city, Shizuoka 426-8677, Japan; b Department of Otolaryngology, Fujieda Municipal General Hospital, 4-1-11, Surugadai, Fujieda-city, Shizuoka 426-8677, Japan; c Department of Anesthesiology, Fujieda Municipal General Hospital, 4-1-11, Surugadai, Fujieda-city, Shizuoka 426-8677, Japan; d First Department of Surgery, Hamamatsu University School of Medicine, 1-20-1 Handayama, Higashi-ku, Hamamatsu city, Shizuoka 431-3192, Japan.

**Keywords:** Airway obstruction, Acute empyema, Pyothorax, Tracheal stenosis

## Abstract

**Introduction:**

Inhalation injury is a major complication of fire accidents. Delayed onset of tracheal stenosis is one of the chronic complications of inhalation injury.

Here, we report a case of acute empyema as a complication of inhalation injury.

**Presentation of case:**

A 38-year-old-man who underwent a tracheostomy following an inhalation injury when he was 25-years of age was admitted with a diagnosis of right-side pyothorax. We attributed the pyothorax to insufficient bronchial toilet secondary to preoperative tracheal stenosis and tracheal mucosal damage as a complication of inhalation injury, as confirmed using laryngofiberscopy.

Conservative therapy was insufficient, therefore, surgical drainage was performed. At the time of surgery, following general anesthesia induction, the insertion of a single-lumen tube was difficult owing to severe tracheal stenosis. As a result, we performed an emergency tracheostomy followed by empyema curettage.

**Discussion/conclusion:**

Tracheal stenosis due to tracheal basal membrane injury and mucosal membrane injury resulted in sputum clearance disorder. These changes led to pyothorax.

Preoperative airway safety should be carefully planned when operating on patients with tracheal stenosis.

## Highlights

• Pyothorax from inhalation injury treated with surgical curettage and tracheostomy
• Tracheal stenosis and injury to the tracheal mucosal membrane caused dysphonia.
• Emergent tracheostomy was performed owing to difficulty with oral intubation.
• Preoperative airway safety planning is essential for tracheal stenosis patients.

## 1 Introduction

Inhalation injury is a major complication of fire accidents. Airway obstruction due to edema and bronchopneumonia are major complications during the acute phase [1]. Conversely, delayed onset of tracheal stenosis is one of the chronic complications of inhalation injury [2].

Here, we report a case of acute empyema related to complications associated with inhalation injury. This work has been reported in line with the SCARE criteria [3].

## 2 Presentation of case

**Fig. 1:**
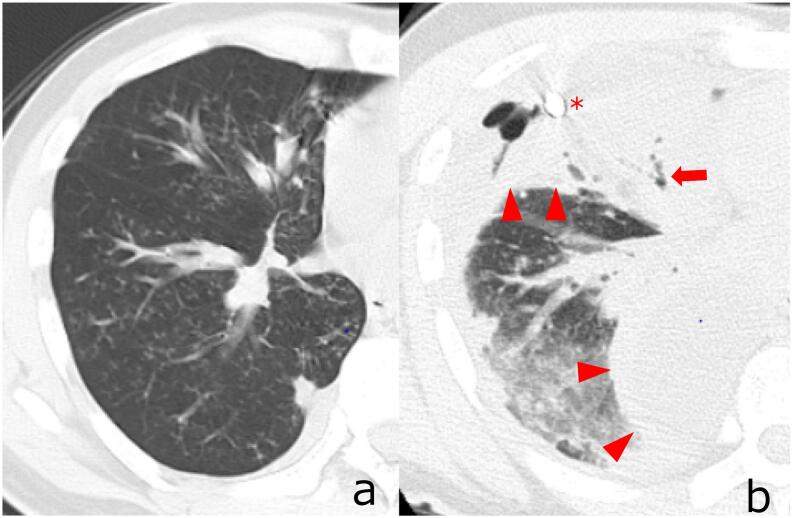
Chest CT before and after admission. a: Chest CT showing diffuse panbronchiolitis 2 weeks before admission. b: Chest CT showing pneumonitis in the right lung. The bronchioles were obstructed by phlegm (arrows). Multifocal pleural effusion is observed in the right thoracic cavity (arrowhead). A chest drainage tube is inserted into the ventral space (asterisk).

A 38-year-old man with a chronic cough presented to our hospital 2 weeks before admission. When he was 25-years old, he suffered an inhalation injury due to a fire at his home, which resulted in glottic and tracheal stenosis requiring a tracheostomy and bilateral glottoplasty. Subsequently, a tracheostomy closure was performed. He had no additional medical history. He admitted to chronic coughing for the past year. The patient had chronic aspiration due to vocal cord palsy. Chest computed tomography (CT) revealed diffuse pan-bronchiolitis (Fig. 1a). As the patient's general status was stable, he initially received antibiotic therapy (erythromycin 400 mg/day). He was referred to our emergency department 2 weeks later. He complained of fever and breathing difficulty.

**Fig. 2:**
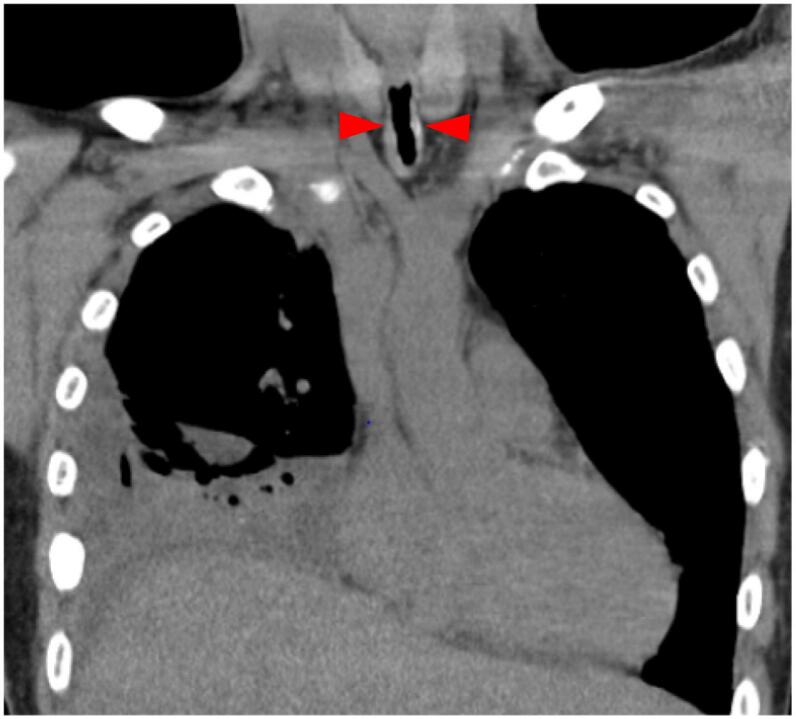
Chest CT of the narrowest area of trachea. The diameter of tracheal narrowing area was 6 mm at preoperative CT.

The patient presented with sputum production and wheezing. He was administered oxygen via nasal canulae at 3 L/min. Blood tests revealed neutrophilic leukocytosis and elevated C-reactive protein levels. A chest CT scan showed pneumonia and multifocal pleural effusion in the right thoracic cavity that was not present 2 weeks prior to admission (Fig. 1b). His chest CT also showed tracheal stenosis (Fig. 2). The diagnosis was acute empyema secondary to worsening pneumonia. We assessed sputum expectoration disorders that led to a spectrum of respiratory infections, from pneumonia to pyothorax. He initially received antibiotic therapy (piperacillin tazobactam 18 g/day and azithromycin 500 mg/day) and chest tube drainage; however, the therapeutic effect was insufficient. Subsequently, surgical drainage was performed under general anesthesia.

We planned to perform tracheostomy after curettage of the pyothorax. We concluded that a tracheostomy was necessary because tracheal stenosis and insufficient bronchial toilet caused the pyothorax, making postoperative extubation difficult.

**Fig. 3:**
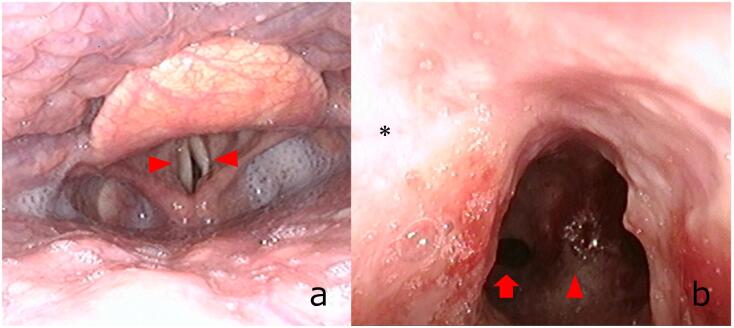
Fiberscope before operation. a: Preoperative fiberscopy showed insufficient expansion of the bilateral vocal cords upon inhalation. The vocal cords are towed by glottoplasty (arrowhead). b: The tracheal mucosa was desquamated and scarred. Tracheal stenosis is observed (asterisks). The carina (arrowhead) and left main bronchus (arrow) are shown.

We performed preoperative pharyngeal fiberscopy to assess airway stenosis. Fiberscopy revealed insufficient expansion of the bilateral vocal cords after bilateral glottoplasty (Fig. 3a). The tracheal mucosa was desquamated and scarred. Tracheal stenosis was confirmed (Fig. 3b). The area of damaged mucosa was broad, so we thought this change was occurred as a result of inhalation injury.

The diameter of the narrowed section of the trachea was 6 mm as detected using the preoperative CT (Fig. 2). Considering the diameter of the narrowest area, we initially planned to insert a single-lumen tube and establish isolated lung ventilation with a tracheal blocker.

After induction of general anesthesia, oral intubation proved difficult; therefore, tracheostomy was preceded by ventilation with a laryngeal mask (i-gel4 © Japan Medical Next) in the supine position. Empyema curettage was performed after tracheostomy (BLU select suction aid 7.0 mm Internal Diameter ©Smith Medical Japan) with the patient in the left decubitus position. Under bilobar ventilation, an approximately 15-cm skin incision was placed along the 6th intercostal space and a thoracotomy was performed. We confirmed reactive pleural effusion and pus in the thoracic cavity, and performed as much curettage as possible. The intraoperative sputum and pleural effusion cultures were both negative. The postoperative course was uneventful, without pyothorax recurrence or any tracheostomy site problems. The patient was discharged 24 days postoperatively. He retained tracheostomy because tracheostomy closure seemed to be difficult owing to dysphagia. He returned to the outpatient clinic once a month for 6 months after surgery for tracheal canulae exchange.

## 3 Discussion

Airway obstruction can be fatal in patients with burns, which may be due to airway edema in the acute phase [1]. In the presence of an intact basal cell layer, early repair can be quickly accomplished during clinical and experimental observations. If the basal membrane is destroyed by an inhalation injury, delayed repair may result in granulations, cicatrization, and stenosis in the chronic phase [4]. Mucosal membrane injury leads to decreased motility of tracheal ciliated epithelium and can result in sputum clearance disorder [5].

In the present case, the patient developed chronic tracheal and glottal stenosis as a complication of inhalation injury.

Additionally, the patient experienced chronic aspiration due to vocal cord palsy. Chest CT revealed bronchial sputum accumulation and bronchial stenosis. The patient developed empyema due to disordered expectoration, which developed as a result of airway stenosis and mucosal membrane injury. This was a rare case as the development of the pyothorax was unique. There are few case reports of pyothorax related to airway stenosis, tracheal mucosal injury, and chronic aspiration due to inhalation injury.

Regarding the operation procedure, we initially planned empyema curettage under oral intubation. Initially, we planned to intubate the patient with a single-lumen tube and establish isolated lung ventilation using a tracheal blocker. However, after the induction of general anesthesia, we re-observed airway in detail, and determined that it would be difficult to insert a single-lumen tube due to tracheal stenosis. Even if intubation was possible, airway narrowing due to edema could occur. Therefore, we performed a tracheostomy under laryngeal mask ventilation. Preoperative airway safety should be carefully planned when operating on patients with tracheal stenosis.

Tracheal resection and anastomosis could be a surgical option for another treatment of tracheal stenosis [6]. However, in this case, we did not choose either of these because the cause of pyothorax was tracheal stenosis and insufficient bronchial toilet. Tracheostomy was necessary for this patient due to sputum clearance disorder as a result of tracheal mucosal membrane injury.

## 4 Conclusion

This case of pyothorax in a patient with airway stenosis was unique because the development of the condition resulted from bronchial sputum accumulation, bronchial stenosis and chronic aspiration due to damage caused by a trauma that occurred 13 years previously.

Preoperative airway safety should be carefully planned when operating on patients with tracheal stenosis.

## Consent for publication

Written informed consent was obtained from the patient for publication of this case report.

## Ethical approval

This study was exempted from approval in our institution because patient consent was obtained and all identifying personal health information was removed from the presentation.

## Funding

Not applicable.

## Author contribution

Asuka Uebayashi wrote this paper. All authors read and approved the final manuscript.

## Guarantor

Asuka Uebayashi.

## Research registration number

Not applicable.

## Conflict of interest statement

The authors declare that they have no competing interests.
